# Spectroscopic Investigation of Tomato Seed Germination Stimulated by *Trichoderma* spp.

**DOI:** 10.3390/biology13050340

**Published:** 2024-05-13

**Authors:** Igor Vukelić, Danka Radić, Ilinka Pećinar, Steva Lević, Daniela Djikanović, Ksenija Radotić, Dejana Panković

**Affiliations:** 1 Faculty of Ecological Agriculture, Educons University, Vojvode Putnika 87, 21208 Sremska Kamenica, Serbia; igor.vukelic@educons.edu.rs; 2 Institute of General and Physical Chemistry, Studentski trg 12/V, 11000 Belgrade, Serbia; dradic@iofh.bg.ac.rs; 3 Faculty of Agriculture, University of Belgrade, Nemanjina 6, 11080 Belgrade, Serbia; ilinka@agrif.bg.ac.rs (I.P.); slevic@agrif.bg.ac.rs (S.L.); 4 Institute for Multidisciplinary Research, University of Belgrade, Bulevar despota Stefana 142, 11060 Belgrade, Serbia; danielle@imsi.rs (D.D.); xenia@imsi.bg.ac.rs (K.R.); 5 Julius Kuehn Institute, Institute for Resistance Research and Stress Tolerance, Erwin Baur Strasse 27, 06484 Quedlinburg, Germany

**Keywords:** symbiotic endophyte, *Solanum lycopersicum* L., cell wall modification, plant growth promotion, radicles

## Abstract

**Simple Summary:**

Fungi of the genus *Trichoderma* have a number of positive effects on plants that are manifested in the stimulation of plant growth and tolerance to abiotic and biotic stress factors. Our findings provide evidence for the swift response of tomato seeds to *Trichoderma* spp. exposure under in vitro conditions. The response was examined after 48 h of germination, before any physical contact was established between *Trichoderma* spp. and tomato seeds. Spectroscopic methods were used to reveal the nature of tomato seed germination stimulation by *Trichoderma*. Induction of the higher synthesis of phenolic compounds through plant specific responses within seed radicles was indicated. Observed differences between treatments were predominantly based on modifications in the pectin content in the middle lamella, as well as alterations in hemicelluloses and xyloglucan within the primary cell wall. Significant alterations in the composition of tomato seed radicles treated with *Trichoderma* spp., characterized by changes in total protein and a concurrent reduction in pectin and/or xyloglucan levels were observed. Applied spectroscopic methods are valuable tools to investigate the stimulation of seed germination by symbiotic microorganisms. Characterized by low cost and high measurement speed, they could be used in large-scale selection of the best genotype-strain combinations for better germination.

**Abstract:**

Seed germination is a complex process that can be negatively affected by numerous stresses. *Trichoderma* spp. are known as effective biocontrol agents as well as plant growth and germination stimulators. However, understanding of the early interactions between seeds and *Trichoderma* spp. remains limited. In the present paper, Fourier-transform infrared spectroscopy (FTIR) and Raman spectroscopy were used to reveal the nature of tomato seed germination as stimulated by *Trichoderma*. A rapid response of tomato seeds to *Trichoderma* spp. was observed within 48 h on Murashige and Skoog medium (MS) substrate, preceding any physical contact. Raman analysis indicated that both *Trichoderma* species stimulated phenolic compound synthesis by triggering plant-specific responses in seed radicles. The impact of *T. harzianum* and *T. brevicompactum* on two tomato cultivars resulted in alterations to the middle lamella pectin, cellulose, and xyloglucan in the primary cell wall. The Raman spectra indicated increased xylan content in NA with T9 treatment as well as increased hemicelluloses in GZ with T4 treatment. Moreover, T4 treatment resulted in elevated conjugated aldehydes in lignin in GZ, whereas the trend was reversed in NA. Additionally, FTIR analysis revealed significant changes in total protein levels in *Trichoderma* spp.-treated tomato seed radicles, with simultaneous decreases in pectin and/or xyloglucan. Our results indicate that two complementary spectroscopic methods, FTIR and Raman spectroscopy, can give valuable information on rapid changes in the plant cell wall structure of tomato radicles during germination stimulated by *Trichoderma* spp.

## 1. Introduction

The extensive application of chemical fertilizers and pesticides has led to a decrease in soil fertility, a reduction in soil biota, and the development of resistant pathogens [1]. Consequently, there has been a growing trend in agricultural practices of increasing reliance on biological agents [2]. These agents involve the use of microorganisms or their metabolites to protect seeds, enhance germination, promote plant growth, and effectively manage various pathogens and pests [3,4].

Tomato (*Solanum lycopersicum* L.) is one of the most economically significant vegetables globally, ranking as a major crop after the sweet potato and playing a crucial role in agriculture worldwide [5]. However, tomato seed dormancy frequently leads to germination failure in freshly harvested tomato seeds. The persistence of inactivity in seeds aged up to one year has been reported, thereby imparting significant challenges for tomato production on a global scale [6].

The genus *Trichoderma* is a cosmopolitan filamentous Plant Growth Promoting Fungi (PGPF), with the ability to trigger defense mechanisms in plants [7]. These mechanisms induce resistance against both biotic and abiotic stresses while concurrently facilitating plant growth. *Trichoderma*, as biocontrol agents against biotic stress, utilize antagonistic strategies such as competition [8], parasitism [9], and anti-biosis [10]. *Trichoderma* also help plants in alleviating abiotic stress effects such as drought [11,12,13], salinity [14,15], the presence of toxic metals [16,17], and low temperature [12,18]. The means through which *Trichoderma* stimulate plant growth and yield include the synthesis of phytohormones, its influence on nutrient availability and uptake, and the stimulation of plant growth through secondary metabolites [19,20].

Over the past decade, *Trichoderma* spp. have gained recognition not only as effective biocontrol agents and plant growth stimulators but also as seed germination enhancers [19]. Consequently, treatments applied to seeds can bring about enhancements in seed quality and subsequent plant performance, both in the short term and long term. However, understanding of the early interactions between seeds and *Trichoderma* spp. remains limited [21]. The germination stage is considered the most crucial phase in a plant’s life cycle, as plants are highly susceptible to disease, damage, and environmental stress [22]. During seed germination after prolonged storage or osmotic stress, a pronounced rise in respiratory activity occurs, accompanied by heightened generation of reactive oxygen species (ROS) [23], a common factor that negatively affects plants. Research conducted by [21] revealed that in seeds subjected to prolonged storage or exposed to osmotic stress, subsequent treatment with *Trichoderma* resulted in a reduction in the accumulation of lipid peroxides, which, in turn, had a positive effect on seed germination. The stimulation of seed germination by *Trichoderma* was shown to be closely associated with the production of phytohormones, including gibberellins, brassinosteroids, and indole acetic acid [24,25], as well as auxin accumulation in the roots by Volatile Organic Compounds (VOCs) [26].

It is well documented that Plant—*Trichoderma* interactions are genotype- and *Trichoderma* strain-dependent [27,28]. The aim of this work was to study the short-term effects of five *Trichoderma* species (*T. harzianum*, *T. brevicompactum*, *T. virens*, *T. longibrachiatum*, and *T. citrinoviride*) on the seed germination of two tomato cultivars (Narvik and Gruzanski zlatni). To determine the indicator of tomato seed stimulation by the two best strains, *T. harzianum* and *T. brevicompactum*, a study was conducted utilizing both Fourier-transform infrared spectroscopy (FTIR) and Raman spectroscopy techniques. Fourier-Transform Infrared (FTIR) and Raman spectroscopy are invaluable tools in analyzing data to provide detailed molecular information. Applied together, FTIR and Raman spectroscopy enable studying structural changes in the cell walls of two tomato cultivars in the presence of the two fungi strains. The Raman spectroscopy was applied to monitor cell wall constituents (polysaccharides and lignin); in addition, FTIR spectroscopy provided information on changes in protein content. Raman and FTIR spectroscopic methods are characterized by low cost and high measurement speed, with little or no need for sample preparation, in comparison to standard biochemical methods. Here, we show that they are valuable tools in contributing to a better understanding of plant–microbe interactions.

## 2. Materials and Methods

### 2.1. Fungal Strain and Growth Condition

The species used in this experiment, *T. harzianum* SZMC 20660 (T4), *T. brevicompactum* SZMC 22661 (T9), *T. virens* SZMC 22659 (T7), *T. longibrachiatum* SZMC 22664 (T11), and *T. citrinoviride* SZMC 22668 (T13) (deposited at Educons University, Faculty of Ecological Agriculture, in Serbia and the University of Szeged, Department of Microbiology, in Hungary) were isolated from the A horizon (5–30 cm) of agricultural soil (Table 1). Species identification was based on their internal transcribed spacer (ITS) and TEF1-α sequences [29,30]. Pure cultures of five *Trichoderma* isolates were transferred from 20% glycerol solution stored at −20 °C to potato dextrose broth (PDA) medium and were incubated at 25 °C in the dark. Four-day-old *Trichoderma* isolates were transplanted from PDA (5 mm diameter cut discs) to the center of a 9 cm diameter Petri dish containing Murashige and Skoog medium (MS) [31] (MS Basal Salts Mixture, M5524 Sigma Aldrich, St. Louis, MI, USA). Also, 30 seeds were distributed around the rim of each Petri dish for each treatment.

### 2.2. Plant Material and Growth Conditions

Two medium-late tomato cultivars, Gruzanski zlatni (GZ) and Narvik (NA), often used in industrial production due to the good quality of the fruit and resistance to diseases, were investigated. GZ is resistant to diseases *Fusarium oxysporum* f. sp. *Lycopersici*, *Verticillium albo-atrum*, and *Phytophthora infestans*, while NA is resistant to *Fusarium oxysporum* f. sp. *Lycopersici* and *Verticillium albo-atrum* [32,33]. The seeds of the tomato varieties were obtained from the Institute for Vegetable Crops, Smederevska Palanka, Serbia, while the germination of seeds and the collection of plant material was conducted at Educons University, Sremska Kamenica, Serbia.

**Figure 1:**
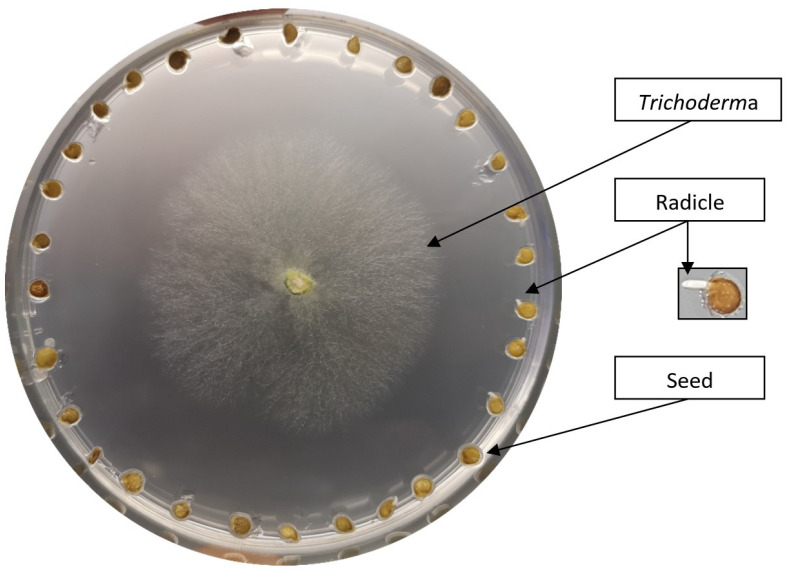
Tomato seed (GZ) cultivated in the presence of *T. harzianum* on MS medium.

Tomato seeds were sterilized in 2% NaClO for 20 min and then washed with distilled water. The seeds were germinated in a Petri dish (Ø 15 cm) on a 0.5 × MS medium for 48 h in darkness at 25 °C. Tomato seeds of GZ and NA were co-cultured with five *Trichoderma* species. The impact of *Trichoderma* spp. on the germination process of two tomato cultivars was investigated, over 48 h, before direct physical contact between the *Trichoderma* and the tomato seed was established (Figure 1). Research was conducted on over 10,000 tomato seeds, comprising approximately 5000 GZ and 5000 NA seeds.

### 2.3. Scanning Electron Microscope (SEM) Measurements

According to a study [34], SEM is a suitable method for imaging fungi on a plant sample. Surface imaging of the epidermis and cross-sectional imaging of the radicle of tomato seeds were performed using a scanning electron microscope (SEM) precisely 48 h after sowing the seeds and *Trichoderma* on the MS medium, before establishing physical contact between the fungus and the seed. Additional recordings were taken after 48 h to illustrate the appearance of the contact between the fungus and the plant. The SEM (Tescan Vega TS 5130 MM, Brno, Czech Republic) had a BSE detector (back-scattered electrons) and EDS system (energy dispersive spectroscopy) (“INCAPentaFETx-3” detector, Oxford Instruments, Boston, MA, USA). The prepared samples were observed under magnifications of 200×, 500×, 1000×, 2000×, and 5000×, at a beam voltage of 20 kV.

### 2.4. Raman Spectroscopy Measurements

This method was used to monitor hemicelluloses, xylan, pectin, and lignin as the cell wall constituents. Emerging radicles of tomato seeds (length ~4 mm), co-cultured with two strains that are most effective in stimulating the germination of seeds, either *T. harzianum* or *T. brevicompactum*, were cut longitudinally (thickness of 70 µm) at room temperature and were recorded using an XploRA Raman spectrometer from Horiba Jobin Yvon. For the Raman spectra, there were 4–6 biological replicates for the NA sample type and 9–10 replicates for GZ samples. Raman spectra were recorded using the laser at a wavelength of 785 nm (maximum output power of 125 mW) equipped with 600 line/mm grating. Spectra were acquired by applying an exposure time of 10 s and scanning the sample 10 times, using a 100% filter. The spectral resolution was 3 cm^−1^, and calibration was checked by a 520.47 cm^−1^ line of silicon. The spectral range in the interval from 800 to 1800 cm^−1^ was analyzed. Four to ten spectra were collected and averaged for each sample.

### 2.5. PCA of Raman Spectra

The outcomes of Raman spectroscopy were subjected to Principal Component Analysis (PCA). PCA was carried out on data smoothed, baseline-corrected, and normalized by the highest intensity band and using spectral regions from 800 to 1800 cm^−1^. The spectra preprocessing was realized using Spectragryph software (Oberstdorf, Germany, v1.2.8.) [35]. Spectra were baseline-corrected using Savitzky–Golay filters with 7 points, and a second-order polynomial function was used for spectra smoothing. PCA was performed using PAST software (Oslo, Norway, v4.03) [36]. PCA was performed using 4 to 10 samples of radicles per treatment.

### 2.6. Fourier-Transform Infrared Spectroscopy (FTIR) Measurements

This method was used to monitor the protein content, carbohydrates, and cell wall constituents. The influence of *Trichoderma* spp. on the germination process of tomato seeds was determined using an FTIR spectrophotometer (IRAffinity-1, Shimadzu, Japan). Radicles of tomato cultivars GZ and NA, grown under control conditions as well as in co-culture with the two most perspective strains, either *T. harzianum* or *T. brevicompactum,* were collected from MS media. Radicles (length ~4 mm) were dried at 60 °C for five days, after which they were homogenized. From each treatment, 250 mg of dry material (around 1530 radicles) was collected. Sample analysis was conducted utilizing the KBr tablet technique [37] (1 mg of sample in 200 mg of KBr), encompassing a wavelength range of 4000–600 cm^−1^, 100 scans per spectrum, and a resolution of 4 cm^−1^. Several independent experiments of seed germination and *Trichoderma* growth were performed to prepare three pooled samples per treatment, which were recorded.

### 2.7. Statistical Analysis

To determine statistically significant differences (*p* < 0.05) in the germination rate of tomato seeds under cocultivation with *Trichoderma* spp., a one-way analysis of variance (ANOVA) employing Tukey’s test was conducted. The analysis was executed utilizing GraphPad Prism software version 6.01 (GraphPad Software, San Diego, CA, USA).

## 3. Results

### 3.1. Scanning Electron Micrography (SEM)

**Figure 2:**
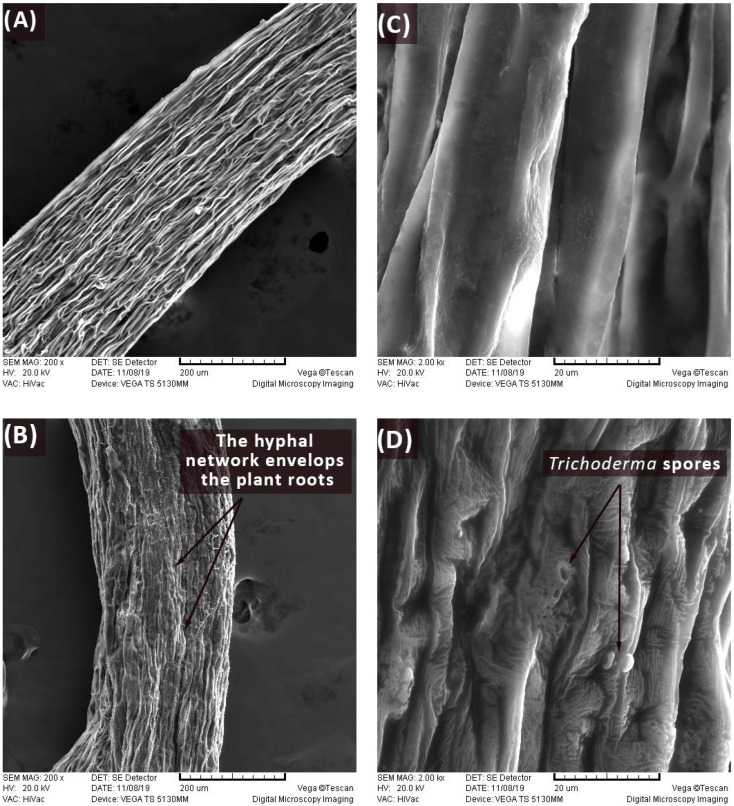
Plant radicles imaged with a scanning electron microscope. GZ tomato radicle epidermis surface in control condition at 200× magnification (**A**) and at 2000× magnification (**C**); GZ tomato radicle epidermis surface after contact with *T. brevicompactum* at 200× magnification (**B**) and at 2000× magnification (**D**). Similar results were obtained for another tomato variety Narvik (not presented).

Scanning electron micrography was conducted to check the presence of physical contact between the *Trichoderma* spp. and the tomato seed radicle during the first 48 h of the co-culture. When radicle samples were collected for Raman and FTIR analysis (Figure 2B), besides the absence of physical contact, it was observed that the radicles in the co-cultures of *T. harzianum* and *T. brevicompactum* were larger and that the zone of the primary bark seen in the longitudinal section was less wrinkled (Figure 2A,C). After 48 h in the co-culture, physical contact was established between *Trichoderma* spp. and the tomato seed radicle. This interaction was visually evidenced in the micrographs, which distinctly depict the emergence of a branched network of hyphal cells accompanied by the presence of spores (Figure 2B,D).

### 3.2. Stimulating In Vitro Tomato Seed Germination with Trichoderma spp.

**Figure 3:**
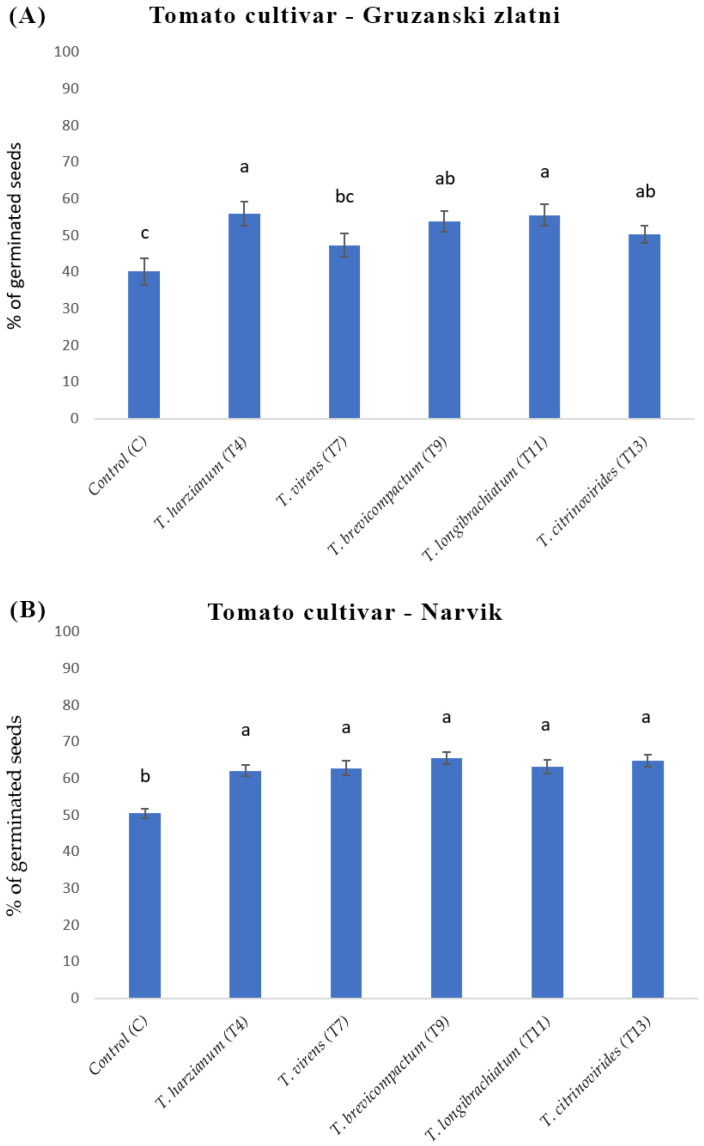
Percentage of germinated tomato seeds, after 48 h on MS media under different treatments: control (C), *T. harzianum* (T4), *T. virens* (T7), *T. brevicompactum* (T9), *T. longibrachiatum* (T11), and *T. citrinoviride*s (T13). (**A**) Percentage of germinated GZ seeds; (**B**) Percentage of germinated NA seeds. One-way ANOVA and post hoc Tukey’s test were used for statistical analysis of data. Values marked with the same letter are not statistically significantly different according to Tukey’s test (*p* < 0.05). The error lines represent ± standard deviation of the mean. Different letters (a, b and c) indicate statistically significant differences according to Tukey’s test (*p* < 0.05). For each treatment, 28 Petri dishes of 30 seeds each were analyzed (*n* = 28).

The influence of five *Trichoderma* species on the germination of two tomato varieties, GZ and NA, was investigated in vitro. Tomato seeds were germinated for 48 h in co-culture with *T. harzianum*, *T. brevicompactum*, *T. virens*, *T. longibrachiatum*, and *T. citrinoviride* on MS media. The highest stimulation of germination observed for the *T. harzianum* and *T. longibrachiatum* treatment of GZ was 15.7% and 15.4%, respectively. *T. brevicompactum* and *T. citrinoviride* stimulated germination for 13.6% and 10.2%, respectively, in comparison to the control. Only the *T. virens* exhibited no statistically significant enhancement in seed germination compared to the control (Figure 3A).

In comparison to control values, the highest stimulation of NA seed germination was observed for *T. brevicompactum* (15%). The stimulation with other *Trichoderma* species ranged from 11.62% (*T. harzianum*) to 14.31% (*T. citrinoviride*) (Figure 3B).

### 3.3. Determination of Changes in the Cell Wall Using Raman Spectroscopy

**Figure 4:**
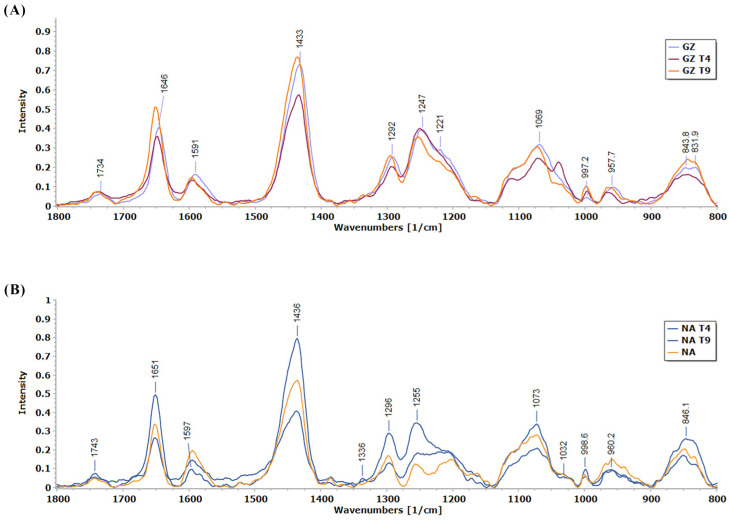
Averages of normalized Raman spectra of two tomato cultivar radicles GZ and NA in control conditions or in co-culture with either *T. harzianum* or *T. brevicompactum*. (**A**) Spectra of the Gruzanski zlatni cultivar radicles (GZ—control; GZT4—*T. harzianum*; GZT9—*T. brevicompactum*); (**B**) Spectra of the Narvik cultivar radicles (NA—control; NAT4—*T. harzianum*; NAT9—*T. brevicompactum*).

The effect of T4 treatment on the cell wall constituents was less pronounced in the NA tomato variety compared to the effects of the T9 treatment (Figure 4). The most pronounced was the effect of the T9 treatment on the spectral region 1200–1260 cm^−1^, indicating that this treatment affected content of hemicelluloses, mostly based on xylan. Also, T9 induced an increase of the protein band at around 1550 cm^−1^, which is not well pronounced in the Raman spectra but is pronounced in the FTIR spectra (Figure 4).

Based on the Raman spectra, it is obvious that *Trichoderma* T4 had a more pronounced effect on the cell wall structural components as compared to the effect of T9 (Figure 4). This influence is most expressed in the three spectral regions. The band 830–850 cm^−1^ related to CH in the lignin monomers’ ring is more pronounced in the T4 treatment and has a different structure compared to the corresponding bands in the control and T9 treatment. The bands at 1040 and 1070 cm^−1^ originate from arabinan-based polysaccharides (arabinan, arabinoglucuronoxylan+galactoglucomannan), while the 1070 cm^−1^ band may originate from xyloglucan. Their ratio is opposite in the spectra of the T4 treatment compared to the control and T9. These differences indicate that arabinan-based hemicelluloses and xyloglucan are significantly targeted in the T4 treatment. The region 1100–1150 cm^−1^ is more expressed and differently structured in the T4 treatment compared to the control and T9. Some smaller changes are observed in the region 1200–1300 cm^−1^, indicating the influence of both T4 and T9 on xylan-based hemicelluloses. The band with the highest intensity in all samples is in the spectral region from 1433 to 1437 cm^−1^ and might be related to the phenolic compounds in tomato radicles (Figure 4). The region 1594–1596 cm^−1^ is characteristic of C=O stretch in the conjugated aldehydic structures in lignin. This band is most pronounced in T4-treated plants, indicating the highest content of such type of structure in lignin of this sample. The T4 had some effect on the 1650 cm^−1^ band, most possibly related to proteins (Table 2).

### 3.4. PCA Analysis of Raman Spectra of Two Tomato Cultivar Radicles in Co-Culture with Either T. harzianum or T. brevicompactum

**Figure 5:**
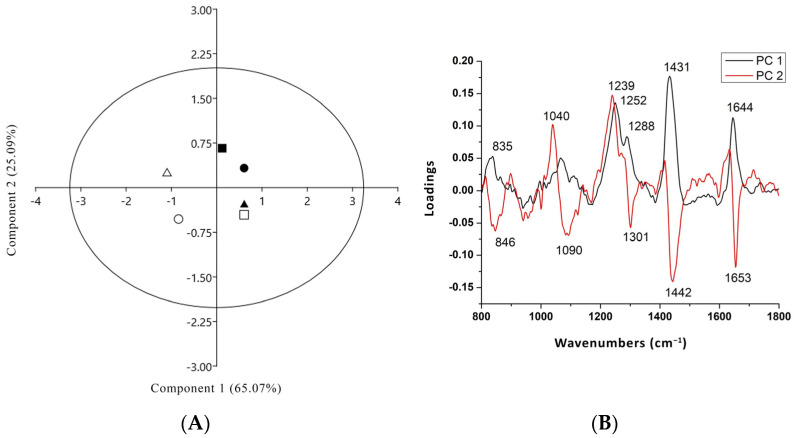
(**A**) Score plot of the first principal component (PC1) versus the second principal component (PC2) of the tomato radicle from two cultivars (GZ and NA) treated with *T. harzianum* (T4) and *T. brevicompactum* (T9). (**B**) Loading plot corresponding to PC1 and PC2 responsible for the differences between the cultivars. N—closed cycle, NAT4—closed square, NAT9—closed triangle, GZ—open cycle, GZT4—open square, and GZT9—open triangle.

Multivariate analysis, based on PCA, was applied for analysis of the Raman spectra of the radicles from two tomato cultivars in co-culture with *T. harzianum* or *T. brevicompactum*. Figure 5 presents the scores and loading plots of the PCA. Figure 5A highlights the good separation into two different groups, where the first and second principal components described 90.17% of data variance. The score plot of PC1 versus PC2 (Figure 5) shows a good separation between the tomato radicle of cultivar Narvik in the control condition and that treated with *T. harzianum* and *T. brevicompactum* (NAT4 and NAT9), and the tomato cultivar Gruzanski zlatni radicle treated with *T. harzianum* (GZT4) was clearly separated from the tomato radicle in the control cultivar (GZ) and the cultivar treated with *T. brevicompactum* (GZT9). For this purpose, loadings for each PC were analyzed to identify which bands had the most influence, such that it was possible to determine which main chemical constituent had the most influence, since each main chemical constituent was represented by multiple bands in the Raman spectra.

The loading plot of the PC1 (Figure 5B) displays the loadings responsible for the separation in the tomato cultivar GZ in co-culture with *T. brevicompactum* (GZT9) and *T. harzianum* (GZT4) from the control tomato radicles (NA) and those in co-culture with *T. harzianum* and *T. brevicompactum* (NAT4, NAT9) as well as GZ. PC1 typically explains the majority of variability (65.08%); therefore, the bands in the spectral range 800–1800 cm^−1^ are primarily assigned to lignin, hemicelluloses including xylan (e.g., broad bands at 835, 1040, 1239–1280 cm^−1^), phenols (1434 cm^−1^), and Amide I of proteins (the loadings at 1644 cm^−1^). The most prominent band, which is responsible for the differentiation among the samples, comes from a broad band position at 1252 cm^−1^, possibly due to the C-O stretch, C-H, and/or C-O-H bending in hemicellulose or pectic acid, as well as the band for the (O-C-O) skeletal mode of Arabian polysaccharides at 1040 cm^−1^.

The results show that the GZ samples are well separated along PC1, while the N samples are separated along PC2. The loading plot of the PC2 (Figure 5B) displays the bands responsible for differences between different NA and GZ samples. The most prominent bands responsible for the differentiation among the N samples come from hemicelluloses including xylan (1040, 1239–1280 cm^−1^), phenols (1442 cm^−1^), and Amide I (1653 cm^−1^). This suggests that in both GZT4 and NAT4, there is a higher content of hemi-celluloses, xylan, and phenolics than in the control and T9 treatment.

### 3.5. FTIR Characterization of Cell Wall Modifications

**Figure 6:**
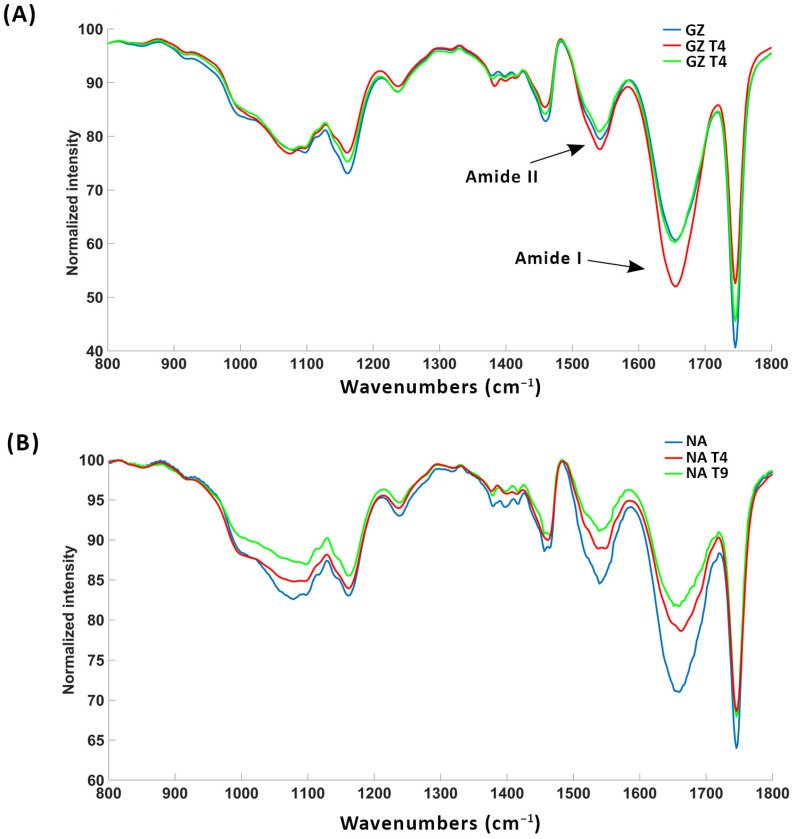
FTIR spectra of homogenized Gruzanski zlatni (**A**) and Narvik (**B**) radicles under different treatments: control (GZ, NA), seeds treated with *T. harzianum* (GZT4, NAT4), seeds treated with *T. brevicompactum* (GZT9, NAT9).

Within the polysaccharide-associated spectral regions, substantial distinctions between the control and treatment samples are generally absent, except for a notable deviation at 1155 cm^−1^ (Figure 6). This particular peak at 1155 cm^−1^ corresponds to the glycosidic vibration in polysaccharides, most probably in pectin (Table 2). The intensity of the peak at 1155 cm^−1^ is the highest in the control, which indicates that *T. harzianum* and *T. brevicompactum* contributed to the reduction in pectin content in the seed radicles. The FTIR spectra of radicles of GZ show that the largest changes occur in the protein part of the spectrum (Figure 6). Prominent protein bands are observed, namely Amide I (max. 1650 cm^−1^) and Amide II (max. 1520 cm^−1^) (Table 2), in both tomato varieties (Figure 6). Relative to the control group, the radicles of GZ seeds treated with *T. harzianum* and *T. brevicompactum* exhibit increased peak intensity, suggesting an increase in protein content, while the decrease in protein peak intensities for the radicles of NA seeds indicate a decrease in protein content in this variety. This result shows a different effect of the fungi on the protein metabolism in the two varieties.

## 4. Discussion

The influence of *Trichoderma* on the growth and development of plants, in the absence of physical contact between these entities, was also examined in *Arabidopsis* plants [26]. The authors investigated in vitro stimulation of *T. atrovirides* and *T. virens* on growth in the plants over a period of 3 to 5 days. It was considered that the stimulation occurs as a result of elevated root auxin content, primarily induced by volatile organic compounds (VOCs) dominated by sesquiterpenes. However, in most of the studies, *Trichoderma* was directly applied on plants. The stimulation of the in vitro germination of tomato (*Arka Meghali*) seeds by eight *Trichoderma* species ranged from 5% to 9% [38]. In a study of the germination and growth response to *T. virens* treatment in numerous plant species, stimulations were connected with improved nutrient uptake and the induction of indole acetic acid (IAA). Similar to our results, three *T. harzianum* isolates where shown to stimulate the germination rate of wheat by about 15% after 48 h in vitro [39]. When the experiment was conducted in soil-filled pots, seed germination increased by 45%.

Plant cell walls are very complex and highly variable structures. Their basic structural elements are crystalline cellulose [(1-4)-β-linked glucose] microfibrils embedded in matrix polymers [40]. In the primary cell wall built during early growth, a network is formed together with cellulose, hemicellulose, and pectin [41], with the addition of non-polysaccharide components like proteins, lipids, enzymes, and aromatic compounds [42]. All the different polysaccharides present in the cell walls give rise to the Raman signature of plant cell wall spectra, with partly broadening and overlapping of characteristic bands, but some marker bands for functional groups of pure polysaccharides still exist [41].

The cell walls have a role in seed germination control. It was shown that disruption of genes involved in pectin maturation and hemicellulose deposition strongly influence germination dynamics [43]. In the primary cell wall, the existence of covalently linked pectin to hemicelluloses, especially xylan, is of crucial importance for the cell wall structure [44]. Xyloglucan, a hemicellulose polymer in the primary cell walls, contributes to loosening or stiffening of the wall during cell elongation [45], and its synthesis is under control of a set of enzymes. Based on the vibrational bands characteristic of the glycosidic vibrations of polysaccharide pectin and hemicelluloses xyloglucan and xylan, it can be concluded that in the radicles of *Trichoderma*-treated seed, the content of pectin decreased in both tomato genotypes. The content of xylan increased in Narvik treated with T9, based on vibration at 1230–1270 cm^−1^. The content of hemicelluloses increased in GZ in the presence of T4, based on the band at 1040 cm^−1^. Based on the band at 1596 cm^−1^, in GZ the conjugated aldehydes in lignin increased in the presence of T4, while in Narvik, the trend was opposite. Characteristic bands for cell wall constituents in the spectral region 1730–1750 cm^−1^ (C=O stretching vibration of the ester carbonyl group) are due to the presence of pectin and hemicellulose [46,47,48] and are higher in NA treated with T9. The assignments of the characteristic bands in the vibrational spectra of the seedlings are shown in Table 2.

**Table 2:** Characteristic bands in the vibrational spectra of the tomato seed radicles.

Peak Position (cm^−1^)	Assignment	References
817–832	C-H out of plane in position 2, 5, and 6 of G units (lignin)	[49]
833	ring, pectin	[46]
854	C-H out of plane in position 2, 5, and 6 of G units, aromatic	[49]
921	C-H out of plane; aromatic	[49]
940–960	Pectic polysaccharides	[50]
966	-CH=CH- out-of-plane deformation	[49]
1000–1300	hemicellulose region	[46,47]
1040 + 1070	arabinan polysaccharides (hemicellulose) (arabinan, arabinoglucuronoxylan+galactoglucomannan)	[47]
1070	Xyloglucan	[47,50]
1230–1270	C–O stretchingvibration in O––C–O, in hemicellulose (xylan), pectin	[47,50,51]
1433–1437	C-H in phenols	[50]
1520	Amide II- stretching bands of protein	[40]
1596	C=O stretching (conjugated aldehydes) in lignin	[49]
1650	Amide I- stretching bands of protein	[52]

Studies involving the root systems of spring and winter wheat cultivars in association with *T. cremeum* and *T. atroviride* reveal discernible modifications in the composition of the cell wall [53]. The accumulation of lignin and reorganization of pectin were observed, suggesting the use of these species to protect against pathogens. In the primary cell wall of plants that are in the development phase, there is an increase in the content of cellulose, hemicellulose, and pectin [41]. According to the FTIR bands at 1500–1700 cm^−1^, in GZ, the protein content increased in the presence of T4, while in Narvik, the protein content decreased, especially in the T9 treatment. It was shown that seeds and roots of corn plants treated with *T. lixii* had increased soluble protein content as determined by the Bradford method [54]. Similar results were observed in rapeseed seedlings treated with *T. reesei* using a nitrogen/protein analyzer [55]. During the colonization of plants by *Trichoderma* spp., proteomic changes occurred in the roots and aerial parts of the plant [56]. However, the molecular mechanisms of action of *Trichoderma* on plant root protein content when physical contact is not established are still insufficiently investigated.

*T. reesei* was shown to secrete enzymes like esterase, xyloglucanase, and β-1,4 endoglucanase, capable of breaking down xyloglucan structures [57]. We also showed previously that *Trichoderma* spp. secrete various extracellular enzymes. It is possible that some of these enzymes may migrate through the medium and may affect seed germination [27,30,57]. According to the API-ZIM test, all tested *Trichoderma* spp. isolates exhibited similar high activity in three enzymes: acid phosphatase, naphthol-AS-BI-phosphohydrolase, and N-acetyl-β-glucosaminidase [27,30,58]. Data in the literature indicate that two of the three mentioned enzymes are active during the seed germination process. Acid phosphatases are significant in the metabolic processes of plant germination and maturation [59]. They exhibit heightened activity during germination and facilitate the release of reserve materials for the growing embryo [60]. N-acetyl-β-glucosaminidase exhibits increased activity during the germination of cotton seeds [61] and radishes [62]. Recent research has also suggested its use in agriculture to promote seed germination and provide plant protection [63].

Our results indicate that two complementary spectroscopic methods, FTIR and Raman spectroscopy, can give valuable information on rapid changes in the plant cell wall structure of tomato radicles during germination stimulated by *Trichoderma* spp.

## 5. Conclusions

To elucidate the early indicators of tomato seed stimulation by *Trichoderma* spp., a study was carried out using both FTIR and Raman spectroscopy techniques. The results of this study provide evidence for a rapid response of tomato seeds to *Trichoderma* spp. exposure under in vitro conditions after 48 h on MS substrates, before any physical contact between *Trichoderma* spp. and tomato seeds was established.

Raman analysis reveals that *T. harzianum* and *T. brevicompactum* induce higher synthesis of phenolic compounds through genotype-specific responses within seed radicles. The observed differences between treatments are predominantly based on modifications in the pectin content in the middle lamella, as well as alterations in hemicelluloses and xyloglucan within the primary cell wall. The Raman spectra indicate that the content of xylan increased in NA treated with T9. The content of hemicelluloses increased in GZ in the presence of T4. In GZ, the conjugated aldehydes in lignin increased in the presence of T4, while in NA the trend was opposite. Analysis of the FTIR spectra revealed significant alterations in the protein content of tomato seed radicles treated with *Trichoderma* spp. in both cultivars. The obtained results provide valuable insights into the molecular mechanisms underlying the interaction between tomato seeds and *Trichoderma* spp., contributing to a better understanding of plant–microbe interactions. Further investigation should aim to identify the specific compounds responsible for stimulating seed germination. FTIR combined with proteomics should be used to define early changes in proteins in seed radicles and/or small proteins excreted by *Trichoderma*.

## Figures and Tables

**Table 1:** *Trichoderma* species selected for the experiment.

Genus	Species	Code	No. of Isolates in the SZMC Collection	Accession Number
*Trichoderma*	*harzianum*	T4	22660	KP316448.1
*Trichoderma*	*brevicompactum*	T9	22661	KP316440.1
*Trichoderma*	*virens*	T7	22659	KP316449.1
*Trichoderma*	*longibrachiatum*	T11	22664	KP316444.1
*Trichoderma*	*citrinoviride*	T13	22668	KP316445.1

## Data Availability

Data available on request.
